# THz Fingerprints of Cement-Based Materials

**DOI:** 10.3390/ma13184194

**Published:** 2020-09-21

**Authors:** Jorge S. Dolado, Guido Goracci, Eduardo Duque, Pavel Martauz, Yibing Zuo, Guang Ye

**Affiliations:** 1 Centro de Física de Materiales, CSIC-UPV/EHU, Paseo Manuel de Lardizabal 5, 20018 Donostia/San Sebastián, Spain; Guido_goracci@ehu.eus; 2 Donostia International Physics Center (DIPC), Paseo Manuel de Lardizabal 4, 20018 Donostia/San Sebastián, Spain; eduardo.duque@ehu.eus; 3 Microlab, Section Materials and Environment, Faculty of Civil Engineering and Geosciences, Delft University of Technology (TU DELFT), Stevinweg 1, 2628 CN Delft, The Netherlands; Zuo@tudelf.nl (Y.Z.); G.Ye@tudelft.nl (G.Y.); 4 Povazska Cementaren a.s., Ladce, 01863 Ladce, Slovakia; martauz.p@pcla.sk; 5 Hubei Key Lab of Control Structures, Huazhong University of Science and Technology, No. 28, Nanli Road, Hong-shan District, Wuhan 430068, China

**Keywords:** THz characterization, atomistic modelling, cementitious structure

## Abstract

To find materials with an appropriate response to THz radiation is key for the incoming THz technology revolution. Unfortunately, this region of the electromagnetic spectra remains largely unexplored in most materials. The present work aims at unveiling the most significant THz fingerprints of cement-based materials. To this end transmission experiments have been carried out over Ordinary Portland Cement (OPC) and geopolymer (GEO) binder cement pastes in combination with atomistic simulations. These simulations have calculated for the first time, the dielectric response of C-S-H and N-A-S-H gels, the most important hydration products of OPC and GEO cement pastes respectively. Interestingly both the experiments and simulations reveal that both varieties of cement pastes exhibit three main characteristic peaks at frequencies around ~0.6 THz, ~1.05 THz and ~1.35 THz, whose origin is governed by the complex dynamic of their water content, and two extra signals at ~1.95 THz and ~2.75 THz which are likely related to modes involving floppy parts of the dried skeleton.

## 1. Introduction

Lying at the meeting point of electronic and photonic technologies, the terahertz (THz) region of the electromagnetic spectrum (frequencies from 10^11^ to 10^13^ Hz) has lately attracted significant interest in materials science, communication and biomedical engineering [1]. A key challenge for the THz technology is to find materials with an appropriate response to the THz radiation, as most natural materials demonstrate weak wave-matter interaction at terahertz frequencies. Many fanciful THz devices have been proposed in the state of the art based on metamaterials (engineered subwavelength resonant metallic inclusions on dielectric spacers) for enhancing the response of materials at the THz region. These solutions are indeed a natural extension to the photonic metamaterials proposed in other electromagnetic spectrum frequencies, like the visible [2,3], Infrared (IR) [4,5], millimeter [6,7] and microwave regime [8,9].

Complementary to the previous solutions, it is highly desirable to find materials with an intrinsic reasonable response within the THz region. In this sense, ubiquitous and cheap materials like cementitious materials deserve due attention. In fact, the main hydration products of Ordinary Portland Cements (OPC) and geopolymer (GEO) binders, the C-S-H and N-A-S-H gels respectively, contain many structural features which seem to favor the response to the THz radiation: On the one hand, the C-S-H and N-A-S-H gels are glassy and amorphous. In fact, the structure of C-S-H and N-A-S-H gels resemble distorted tobemorite clays and defective sodalite zeolites respectively, as numerous experiments [10,11,12,13,14,15] and atomistic models [16,17,18,19,20] have verified. In that sense, and though the underlying reason is still under debate, glassy and amorphous materials are known to host an overpopulation of vibrational states at the THz frequencies (the so-called Boson Peaks (BP)) [21,22,23]. Interestingly, recent atomistic simulations have predicted the possible existence of a BP in the C-S-H gel [24], though no experimental proof exists for the time being. In the case of N-A-S-H structures neither experiments nor simulations have paid attention to this interesting aspect. On the one hand, both C-S-H and N-A-S-H gels contain plenty of water molecules (either in the H_2_O or in the OH^−^ form) which strongly interact with light due to their intrinsic electric dipole. In fact, the dielectric loss spectra of bulk water at room temperature exhibits a dominating peak at 0.02 THz (usually ascribed to the α-relaxation) that is flanked by two additional faster processes whose characteristic frequencies lie around 0.3–0.9 THz and 1.3–1.9 THz [25].

Unfortunately, the dielectric response of cement-based materials has not been sufficiently studied. To the best of our knowledge, only a few works have been published to date [26,27,28,29], studying the dynamic of water by broad band dielectric (BDS) spectroscopy at much lower frequencies (10^−2^ to 10^6^ Hz). In this scenario, the present paper aims to report for the first time the state of the art, the experimental and computational dielectric response of cement-based materials to the THz radiation. To this end, THz transmission experiments have been carried out over cement pastes of OPC and Fly Ash (FA) GEO binders. In addition, atomistic simulations have been performed to evaluate the dielectric function of the most important ingredients of OPC and GEO cement pastes, the C-S-H and N-A-S-H gels.

## 2. Materials and Methods

### 2.1. Sample Preparation

#### 2.1.1. OPC Cement Pastes

Starting powders of OPC (CEM I-42.5R) (Povazska Cementaren a.s., Ladge, Slovakia) were mixed with distilled water (Sigma Aldrich, St. Louis, MO, USA) in a water-to-cement ratio of 0.4 by weight. Each specimen was cast in a cylindrical mold (Ø38 × H15 mm) and sealed. After 24 h, the sample discs were moved to a hermetically closed desiccator with 100% RH and kept at 20 °C for 28 days.

#### 2.1.2. GEO Cement Pastes

**Figure 1:**
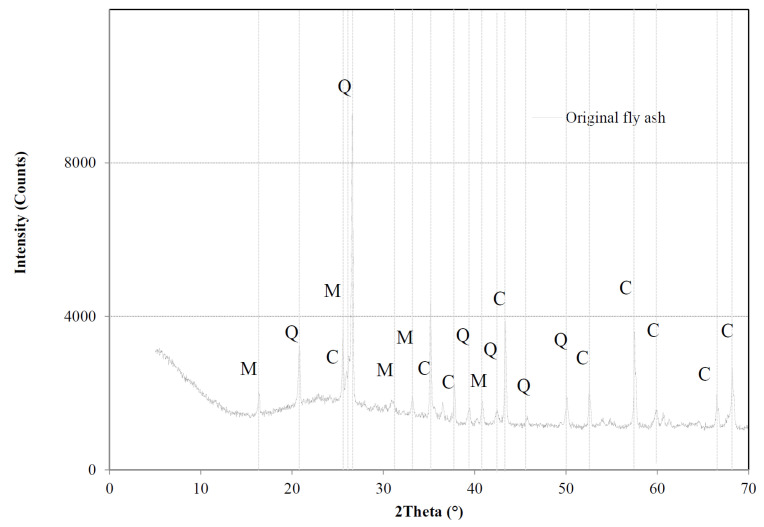
X-ray diffraction analysis of the fly ash, Q = quartz (SiO_2_); M = mullite (Al_4.8_O_9.54_Si_1.2_) and C = Corundum (α-Al_2_O_3_).

Fly ash-based geopolymer cement pastes were used in the experiments. Low calcium fly ash, Class F according to ASTM C 618, from The Netherlands, was used. The chemical composition of the fly ash is given in Table 1. It is noted that the main constituents of the fly ash are SiO_2_ and Al_2_O_3_. Quartz (SiO_2_) and mullite (3Al_2_O_3_ 2SiO_2_) are the main crystalline compounds in the fly ash (Figure 1). The amorphous content of the fly ash, determined by the chemical dissolution treatment (EN 196, Part 2), is 69%. The density and mean particle size of the fly ash is 2.34 g/m^3^ and 21.46 µm, respectively. NaOH activator was prepared by sodium hydroxide (analytical grade > 98%) and distilled water. Then fly ash and NaOH activator were mixed in a commercial Hobart mixer with two minutes low-speed (140 r/min) mixing, followed by two minutes high-speed (285 r/min) mixing. Subsequently the freshly prepared paste was cast into commercial cylinder polyethylene jars (d = 35 mm and h = 70 mm) and vibrated for 30 s on a vibrating table. The water to fly ash mass ratio was 0.35. The samples were cured in a water bath at elevated temperatures (40/60 °C) until test age (28 days).

### 2.2. THz Measurements

The time-domain THz transmission experiments were performed by using a TPS Spectra 3000 spectrometer (TeraView Ltd., Cambridge, UK). Samples were measured covering the spectral range 0.05 to 4 THz at an instrument resolution of ~0.035 THz. A special Teflon sample holder was prepared to investigate powder samples. The use of powder samples makes it impossible to determine with accuracy absolute intensities but avoids the structural changes that can potentially take place when preparing pellets. The size of the powders was below 50 µm to minimize spurious frequency dependent scattering effects in the measured THz window. Each spectrum was collected as 1800 co-added time-domain spectra collected over a period of 1 min.

### 2.3. Atomistic Simulations

#### 2.3.1. Starting Structures

**Figure 2:**
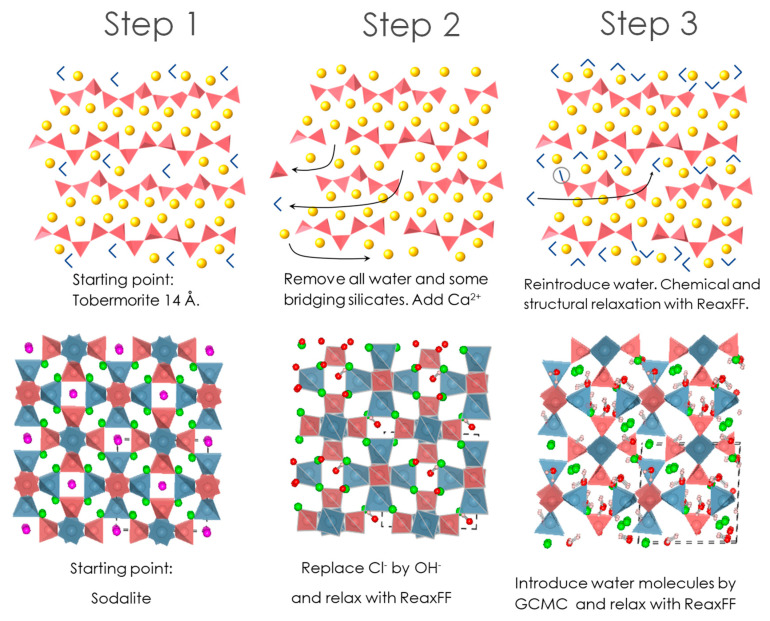
Sketch of the protocol for constructing the C-S-H model (**upper panel**) and N-A-S-H structures (**bottom panel**).

As stated in the Introduction, the key ingredients of OPC and geopolymer-based cement pastes are the C-S-H and N-A-S-H gels, respectively. To simulate the structure of C-S-H the procedure described by Qomi et al. [19], based on an improvement of the original procedure proposed by Pellenq et al. [16] has been employed. A schematic description of the employed protocol for constructing the C-S-H structure is displayed in Figure 2 (upper panel). As such, the structure of Tobermorite 14 (with C/S = 0.83) is taken as the starting point, modifying its structure by firstly removing the water molecules. Afterwards some bridging silicate groups are also randomly removed to get the targeted C/S ratio. Finally, to avoid charge unbalances and get the right water content, some Ca ions and water molecules are randomly added in inter-laminar space. The so obtained structure is finally equilibrated by performing energy minimization and Molecular Dynamic (MD) simulation with the Reax FF [30]. Complete details of the method can be found in Duque [31]. The present study limits the study to the calcium-to-silicon ratio (C/S) 1.67 case, as it is the typical vale found in OPC C-S-H gels. The constructed C-S-H model actually corresponds to a very large system (see Table 2 for the simulation cell parameters) whose exact stoichiometry is (CaO)_254_(SiO_2_)_152_(H_2_O)_306._

On the other hand, a N-A-S-H model has been constructed following a protocol akin to the one recently proposed by Lolli et al. [20], as schematically shown in Figure 2 (lower panel). In particular, the starting structure has been the experimental sodalite structure (Na_8_[Al_6_Si_6_O_24_]Cl_2_) given by Hasan et al. [32] in which we have replaced the Cl atoms with hydroxide ions (OH^−^). After relaxing the structure by using ReaxFF [30], we have applied a Gran Canonical Monte Carlo (GCMC) protocol to introduce water into its structure. To this end a chemical potential of −0.082 eV has been fixed. As a result, the final structure became (Na_2_O)_4_ (Al_2_O_3_)_3_ (SiO_2_)_6_ (OH)_2_·(H_2_O)_9._ Finally, the structure has been relaxed again with ReaxFF, giving the lattice constants and angles disclosed in Table 2.

#### 2.3.2. Dielectric Response Simulations

Computationally speaking, the relevance of this work is surely due to the new methodology disclosed for estimating the dielectric function of cement-based materials. While these sorts of simulations have been already employed in other materials like quartz [33], to the best of our knowledge this is the first time that the dielectric properties of cement-based materials have been simulated. In essence, the underlying idea is that the angular frequency (ω=2πv) dependent dielectric function can be calculated in terms of the atomic vibrations (phonons) and more specifically in terms of the oscillator strength Ω as:

(1) $$\varepsilon_{ij}\left(\omega \right)=\varepsilon_{ij}\left(\infty \right)+\frac{4\pi }{V}\overset{modes}{\underset{m}{\sum }}\frac{\Omega_{ij}^{m}}{\omega_{m}^{2}-\omega^{2}}$$

where the oscillator strength tensor for each vibrational mode m depends on the Born effective charges (*q^B^*) and the eingenvector (*e_ij_*) for that mode according to:

(2) $$\Omega_{\alpha \beta }=\left(\overset{N}{\underset{i}{\sum }}\frac{q_{i\alpha j}^{B}e_{ij}}{m_{i}^{1/2}}\right)\left(\overset{N}{\underset{i}{\sum }}\frac{q_{i\beta j}^{B}e_{ij}}{m_{i}^{1/2}}\right)$$

As in GULP [34] the Born effective charges are not implemented for ReaxFF, these charges have been obtained through the non-reactive force field employed in [18]. To avoid the singularities of Equation (1) a small damping term (δ) of 0.15 THz has been used (i.e., $\omega^{2}\to \omega \left(\omega +i\delta \right)$). For the sake of simplicity, only the diagonal values of the dielectric function matrix have been considered to estimate the values of the dielectric function; i.e., we have taken $\varepsilon \left(\omega \right)=\left(\varepsilon_{xx}\left(\omega \right)+\varepsilon_{yy}\left(\omega \right)+\varepsilon_{xx}\left(\omega \right)\right)/3$ for the real (ε_1_) and imaginary part (ε_2_) of the dielectric function.

Finally, from the knowledge of the dielectric function, the complex refraction index (*n* = *n*_1_ + *i n*_2_) and the absorbance (α) can be obtained from Equations (3) and (4), respectively:

(3) $$\varepsilon_{1}+i\varepsilon_{2}\;=\;{\left(n_{1}+in_{2}\right)}^{2}$$

(4) $$\alpha \left(\omega \right)=\frac{2\omega n_{2}\left(\omega \right)}{c}$$

## 3. Results

**Figure 3:**
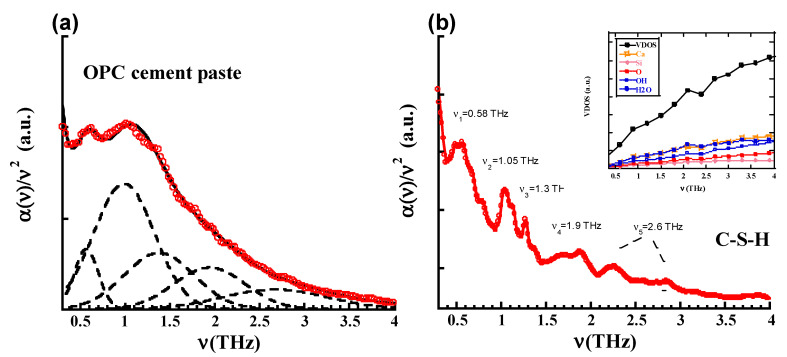
(**a**) Experimental absorbance of the Ordinary Portland Cements (OPC) cement paste, together with the deconvolution of the spectra. (**b**) Computational prediction of the absorbance for the C-S-H model. In the inset the Vibrational Density of States (VDOS) and their projections are displayed.

**Figure A1:**
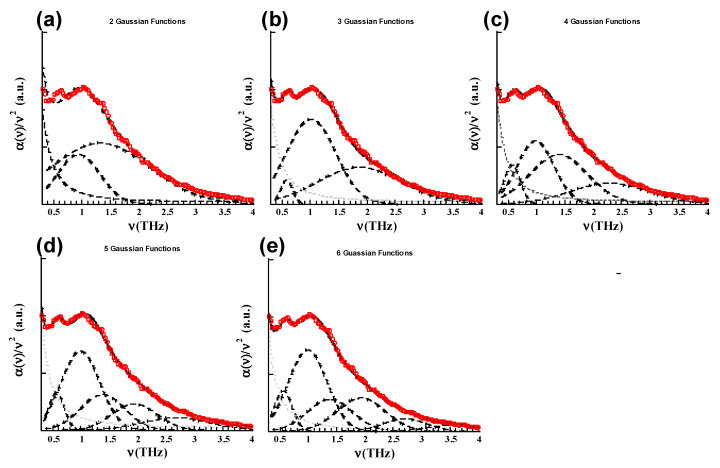
Deconvolution of the absorbance of OPC cement paste spectra with 2 (**a**), 3 (**b**), 4 (**c**), 5 (**d**) and 6 (**e**) gaussians.

**Figure A2:**
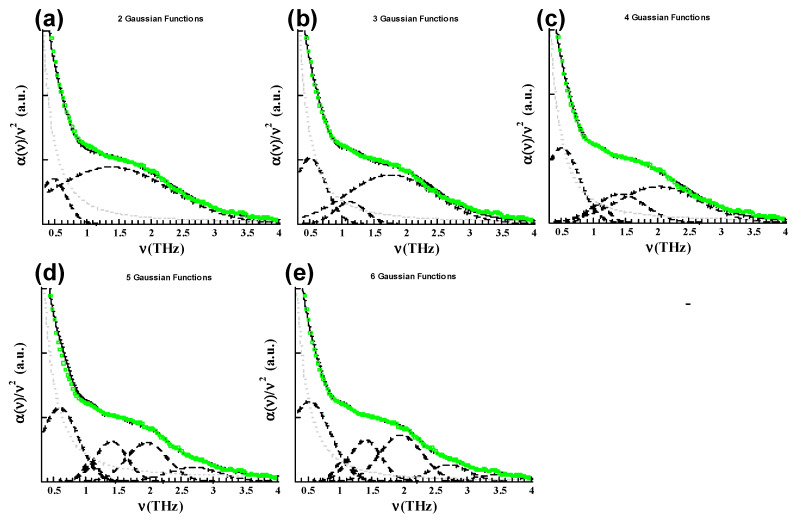
Deconvolution of the absorbance of GEO cement paste spectra with 2 (**a**), 3 (**b**) 4 (**c**), 5 (**d**) and 6 (**e**) gaussians.

Figure 3 displays the experimental absorbance of the OPC cement pastes in the THz regime (Figure 3a) in comparison to the computational prediction of the response of C-S-H gel (Figure 3b). As is customary in the field, the absorbance has been divided by the square of the frequency to take out the normal ~ν^2^ dependence of the vibrational density of the states and highlight the THz response. As absolute values cannot be comparable because cement pastes contain more phases than C-S-H (apart from the experimental problem of using powders, as explained in the Methods section), arbitrary units have been used to compare the spectra. The deconvolution of the experimental spectra has been performed by fitting the data with several gaussians over a 1/ν^2^ background. As shown in the Appendix A (Figure A1 and Figure A2), at least five gaussians are required for an appropriated fitting of the experimental spectra. The positions of these five gaussians (ν_n_) should be understood as the intrinsic THz fingerprints of OPC cement pastes. The values of these frequencies are collected in Table 3. The simulations also recognize several peaks (ν_n_) that can be ascribed as intrinsic THz fingerprints of the C-S-H gel. These values are also reported in Table 3. Note that the experimental and computational frequencies are in good accord, exhibiting noticeable peaks at v_1_~0.60 THz and v_2_~1.05 THz, and minor ones at v_3_~1.35 THz, v_4_~1.95 THz and v_5_~2.75 THz. This last signal extracted from the deconvolution (v_5_~2.75 THz) can be assigned to the two final bumps of the simulations at 2.35 THz and 2.90 THz.

**Figure 4:**
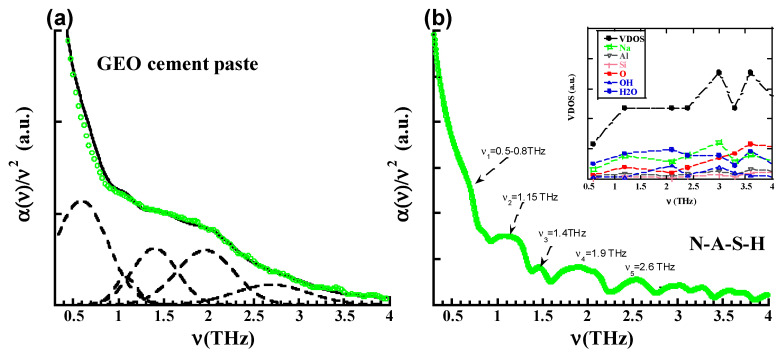
(**a**) Experimental absorbance of the geopolymer cement paste, together with the deconvolution of the spectra. (**b**) Computational prediction of the absorbance for the N-A-S-H model. In the inset the VDOS and their projections are displayed.

The results for the GEO cement paste and the N-A-S-H structure are shown in Figure 4. Figure 4a shows the experimental absorbance together with the deconvolutions and Figure 4b reports the predictions obtained from the atomistic simulations of the N-A-S-H model. In comparison to the case of OPCs, the convolution of the geopolymer spectra exhibits the same THz peaks (v_1_~0.60 THz, v_2_~1.05 THz, v_3_~1.35 THz, v_4_~1.95 THz and v_5_~2.75 THz.), though with a different relative intensity. Again, the simulations are able to capture reasonably well the positions of the peaks. The whole set of frequencies detected experimentally and computationally are collected in Table 3. It is worth noting, nevertheless, that the first peak appears as a diffuse hump in the simulations (0.5–0.8 THz), while in the experiments it is only distinguishable from the background by the deconvolution of the spectra. This is in stark contrast to the case of the OPC paste and C-S-H gel, where this first peak was clearly visible. It is also noteworthy that according to the measurements the relevance of the second and third peaks seems to be inverted in the GEO cement pastes with respect to the OPC cement pastes. Now, the second peak (v_2_~1.05 THz) seems to be much weaker than the third one (v_3_~1.4 THz), while in the OPC cement paste the second one was the stronger one. This pattern is not reproduced in the simulations, where in both the C-S-H and N-A-S-H models, the signal at v_3_~1.4 THz is the weakest one. Finally, the fourth (v_4_~1.95 THz) and fifth peaks (v_5_~2.75 THz) seem to match well with the results found previously in OPC and C-S-H gels. The simulations of N-A-S-H give a slightly better description for the position of these peaks in comparison to the C-S-H case.

To shed light on the origin of these peaks, the Vibrational Density of States (VDOS) and their projections over the atoms/molecules involved have been calculated for the C-S-H and N-A-S-H models. The VDOS at a given frequency ν characterizes the number of phonons having a frequency in the range of n and *ν* + d*ν*. It can be expressed in terms of the total number of modes M and the frequencies of the modes *ν_i_* as

(5) $$g\left(\nu \right)=\overset{M}{\underset{i}{\sum }}\delta (\nu -\nu_{i})$$

The insets of Figure 3b and Figure 4b show the VDOS for the C-S-H and N-A-S.H gel respectively. According to the VDOSs and their projections over the atoms, the low frequency region is clearly dominated by modes involving mainly H_2_O molecules (blue dots) and Ca atoms (orange open squares) in the case of C-S-H gel and H_2_O molecules (blue dots) and Na atoms (green open squares) in the case of N-A-S-H gel. While this dominant role of H_2_O molecules and Ca atoms persists in C-S-H gel along the shown 0.5–4 THz region, beyond this point (though not shown) OH groups (open blue dots) and oxygen atoms (red dots) of the solid C-S-H skeleton start giving substantial contributions. In the case of the N-A-S-H gel, the modes involving the solid N-A-S-H skeleton start earlier (~3 THz), as can be deduced by inspecting the projection over the oxygen atom (red dots).

## 4. Discussion

A simple picture emerges from the inspection of the VDOS and the THz dielectric response. While at high frequencies (>2–3 THz) the observed and predicted low intensity bumps start having contributions coming from the solid skeleton, the five THz peaks observed in the OPC and GEO cement pastes correspond to low frequency water-related vibrations. Thus, it seems clear that the solution trapped in the nano/micro-pores of the cement pastes should have similar vibrational modes, giving rise to an additional contribution to the absorbance intensity. This fact can explain the discrepancies between the intensities measured over the cement pastes and the ones predicted by the simulations for the C-S-H and N-A-S-H gels. Moreover, the positions of the peaks (both measured and predicted) match extremely well with those found in previous studies on confined and hydration water. Very sharp peaks at ~0.6 THz have been found for instance in Molecular Dynamic simulations of supercooled water [35] or in THz transmission measurements over hydrated proteins [36]. Likewise, the second and third peaks at ~1.0 THz and 1.4 THz found in our TH experiments and simulations agree well with the Boson peak of water, since this structural fingerprint has been found at 1.1 THz in protein hydration water [36] and at 1.35 THz in confined water [35,37]. In fact, neutron scattering experiments have recently observed a wide Boson peak at ~1.35 THz for water confined in the porous network of cement pastes [37]. In this sense, it is worth noting that the peaks detected by the THz experiments in OPC cement-based materials seem to emphasize more the signal coming from the “solvation water” (1.1 THz) than the one from the “bulk-like” confined water (1.35 THz). On the contrary, the bulk-like confined water appears as the dominant contribution in GEO matrices. Finally, modes at ~1.95 THz and ~2.75 THz have been previously identified by THz experiments in protein-solvent systems [36,38] and associated to internal side-chain fluctuations of the proteins indirectly connected to the hydration water dynamics. In an analogy to our case, these peaks might be related to modes involving floppy parts of the solid (dried) skeletons, where water molecules are also indirectly affected. In a separate paper we will provide further insight into this issue by studying the influence of the water content on the THz response of OPC and GEO matrices.

So far, this work reports for the first time the state-of-the-art THz measurements over cementitious materials, revealing that this technique can provide valuable structural information. In the range analyzed (0.5–4 THz) five peaks have been found. The first three (v_1_~0.6 THz, v_2_~1.0 THz and v_3_~1.4 THz) are intrinsic fingerprints of the complex water dynamic present in cement-based materials, and the following two (v_4_~1.95 THz and v_5_~2.75 THz) surely relate to modes involving the solid (dried) skeleton. Moreover, it is also the first time that the dielectric properties of cement-based materials have been predicted by atomistic simulations. Considering the approximate nature of the force fields employed, the atomistic simulations have reproduced quite satisfactorily the experimental positions of these peaks. Future research on this topic should explore in greater detail the impact of the water content on the THz response of these materials and extend the scope to other binders like novel hybrid cements (H-CEM) [39], or well established calcium sulphoaluminates (C$A) or calcium aluminates (CA).

## Figures and Tables

**Table 1:** Chemical composition of fly ash.

	Oxide (wt.%)								
	SiO_2_	Al_2_O_3_	CaO	MgO	Fe_2_O_3_	SO_3_	K_2_O	TiO_2_	Other
Fly ash	56.8	23.8	4.8	1.5	7.2	0.3	1.6	1.2	2.8

**Table 2:** Stoichiometry and lattice constants and angles of the studied C-S-H and N-AS-H structures.

	C-S-H	N-A-S-H
Composition	(CaO)_254_ (SiO_2_)_152_ (H_2_O)_306_	(Na_2_O)_4_ (Al_2_O_3_)_3_ (SiO_2_)_6_ (OH)_2_ (H_2_O)_9_
a (Å)	26.083000	9.483393
b (Å)	30.847000	9.11116
c (Å)	25.885000	9.040023
α (°)	90	92.536179
β(°)	90	91.007071
γ (°)	90	88.652042

**Table 3:** Experimental THz frequencies of the main peaks detected in transmission experiments over OPC and geopolymer cement pastes along with the values predicted for C-S-H and N-A-S-H models by atomistic simulations. The uncertainties for the peaks of the fitting scheme have been estimated according to the method explained in Appendix A, while those of the simulations are below the used damping parameter (0.15 THz).

	v_1_ (THz)	v_2_ (THz)	v_3_ (THz)	v_4_ (THz)	v_5_ (THz)
OPC (Exp)	0.58 ± 0.01	1.0 ± 0.01	1.40 ± 0.001	1.95 ± 0.001	2.75 ± 0.01
C-S-H (Sim)	0.58	1.05	1.30	1.90	2.30, 2.85
GEO (Exp)	0.6 ± 0.06	1.0 ± 0.05	1.4 ± 0.01	1.95 ± 0.05	2.75 ± 0.02
N-A-S-H (Sim)	0.5–0.8 hump	1.15	1.4	1.95	2.57

## Appendix A

In order to fit the THz spectra, the undertaken protocol has been the use of several gaussians over a 1/ν^2^ background. A systematic study in terms of the number of gaussians has been performed where the positions and width of the gausssians were freely optimized to fit the spectra. Figure A1 and Figure A2 show respectively the deconvolution of the spectra of OPC and GEO cement pastes in terms of the number of gaussians (N), covering the range from N = 2 to N = 6. The obtained positions of the peaks are disclosed in Table A1. The uncertainty of a given frequency i and a given number of gaussians N (Δν_i_(N)) has been estimated by evaluating (Δν_i_(N) = (|ν_i_(N + 1) − (ν_i_(N)|). These uncertainty values are shown in Table A1 between parentheses. As can be seen in Figure A1c, Figure A2c, at least four gaussians are needed for reasonably good fitting of OPC and GEO samples. However, the inspection of the uncertainties together with the educated information gained from the simulations have made us consider that the use of five gaussians provides a sensible deconvolution over the analyzed frequency.

**Table A1:** Positions of the peaks in terms of the number (N) of used gaussians together with the uncertainty of the positions.

OPC spectra	$\nu_{1}\left(\Delta \nu_{1}\right)$ (THz)	$\nu_{2}\left(\Delta \nu_{2}\right)$ (THz)	$\nu_{3}\left(\Delta \nu_{3}\right)$ (THz)	$\nu_{4}\left(\Delta \nu_{4}\right)$ (THz)	$\nu_{5}\left(\Delta \nu_{5}\right)$ (THz)	$\nu_{6}\left(\Delta \nu_{6}\right)$ (THz)
N = 2	0.92 (0.34)	1.32 (0.34)				
N = 3	0.58 (0.0)	1.01 (0.01)	1.88 (0.48)			
N = 4	0.58 (0)	1.0 (0)	1.40 (0)	2.35 (0.4)		
N = 5	0.58 (0.01)	1.00 (0.01)	1.40 (0)	1.95 (0)	2.75 (0)	
N = 6	0.57	0.99	1.40	1.95	2.75	3.65
GEO spectra	$\nu_{1}\left(\Delta \nu_{1}\right)$ (THz)	$\nu_{2}\left(\Delta \nu_{2}\right)$ (THz)	$\nu_{3}\left(\Delta \nu_{3}\right)$ (THz)	$\nu_{4}\left(\Delta \nu_{4}\right)$ (THz)	$\nu_{5}\left(\Delta \nu_{5}\right)$ (THz)	$\nu_{6}\left(\Delta \nu_{6}\right)$ (THz)
N = 2	0.45 (0.04)	1.44 (0.32)				
N = 3	0.49 (0.02))	1.12 (0.05)	1.82 (0.41)			
N = 4	0.51 (0.09)	1.07 (0.07)	1.41 (0.01)	2.05 (0.1)		
N = 5	0.6 (0.06)	1.0 (0.05)	1.40 (0.01)	1.95 (0.05)	2.75 (0.02)	
N = 6	0.54	1.05	1.41	2.0	2.73	3.53
